# “The health equity curse”: ethical tensions in promoting health equity

**DOI:** 10.1186/s12889-021-11594-y

**Published:** 2021-08-18

**Authors:** Bernie Pauly, Tina Revai, Lenora Marcellus, Wanda Martin, Kathy Easton, Marjorie MacDonald

**Affiliations:** 1 School of Nursing and Canadian Institute for Substance Use Research, University of Victoria, Box 1700 STN CSC, Victoria, BC V8W 2Y2 Canada; 2 Equity Lens in Public Health Project, University of Victoria, Box 1700 STN CSC, Victoria, BC V8W 2Y2 Canada; 3 School of Nursing, University of Victoria, PO Box 1700 STN CSC, Victoria, BC V8W 2Y2 Canada; 4 College of Nursing, University of Saskatchewan, Health Science Building-1A10, Box 6, 107 Wiggins Road, Saskatoon, SK S7N 5E5 Canada; 5 Island Health, 345 Wale Rd, Victoria, BC V9B 6X2 Canada

**Keywords:** Health equity, Relational ethics, Stigma and discrimination, Mental health promotion, Prevention of substance use harms

## Abstract

**Background:**

Public health (PH) practitioners have a strong moral commitment to health equity and social justice. However, PH values often do not align with health systems values, making it challenging for PH practitioners to promote health equity. In spite of a growing range of PH ethics frameworks and theories, little is known about ethical concerns related to promotion of health equity in PH practice. The purpose of this paper is to examine the ethical concerns of PH practitioners in promoting health equity in the context of mental health promotion and prevention of harms of substance use.

**Methods:**

As part of a broader program of public health systems and services research, we interviewed 32 PH practitioners.

**Results:**

Using constant comparative analysis, we identified four systemic ethical tensions: [1] biomedical versus social determinants of health agenda; [2] systems driven agendas versus situational care; [3] stigma and discrimination versus respect for persons; and [4] trust and autonomy versus surveillance and social control.

**Conclusions:**

Naming these tensions provides insights into the daily ethical challenges of PH practitioners and an opportunity to reflect on the relevance of PH frameworks. These findings highlight the value of relational ethics as a promising approach for developing ethical frameworks for PH practice.

**Supplementary Information:**

The online version contains supplementary material available at 10.1186/s12889-021-11594-y.

## Background

Health inequities result from an unequal distribution of the determinants of health, disadvantaging those who lack wealth, power or prestige [1, 2]. Health inequities increase as social position decreases [3, 4]. As social positioning decreases, the higher the concentration of harms from illicit substance use, poor mental health, unmet health care needs, and difficulties accessing health care [5, 6]. These harms often intersect with, and are exacerbated by, the stigma and discrimination associated with drug use, mental illness, poverty, and marginalized social location further affecting health and well-being [7–11]. In such situations, the promotion of health equity raises questions of justice related to the structural conditions that create inequities, such as who has access to resources for health and how structural disadvantages limit access [12].

The twin moral aims of public health are to promote population health and reduce health inequities [13, 14]. Promoting health equity and social justice are part of the ontological foundation of public health (PH) [15–17]. Barrett et al. [18] specifically highlights health equity as an important area of concern for PH ethics. Although there are strong national and international commitments to health equity as a key goal of health systems and services [19, 20], the degree to which organized health systems actually undertake these moral aims is contested [21, 22]. Delivering health services is a complex process with significant political and economic influences and multiple competing demands. However, while PH has an obligation to promote health equity, doing so within a health system that does not prioritize health equity is challenging and a source of ethical tensions that are often not well articulated [23].

In British Columbia, Canada, calls for PH system renewal led to the development of a framework for core functions in public health [24] and later the guiding PH framework [25]. Both the core functions and guiding PH frameworks include a directive to apply a health equity lens in all PH programs. Of the 21 core programs, our PH knowledge user partners identified mental health promotion and prevention of harms of substance use as two important areas to understand and learn about the application of a health equity lens and the ethical issues encountered in promoting health equity. The purpose of this article is to describe and discuss the ethical tensions experienced by PH practitioners with obligations to promote health equity in the core public health areas of mental health promotion and prevention of harms of substance use. We begin with some background on PH ethics followed by a description of the study methodology and then present and discuss findings related to four key ethical issues experienced by PH practitioners.

There are a range of ethical issues identified in the PH literature related to infectious disease control, emergency preparedness, public health communication, cost-effective decision-making and more [18, 26]. For countries without universal healthcare, the lack of universal insurance and the uninsured are key ethical and health equity concerns [27]. Although Canada has a system of universal health care for accessing doctors and hospitals, health inequities in Canada are growing and PH providers often work in health systems that are not aligned with PH values of social justice or address the broader determinants of health [28–31].

Historically, bioethics has focused on individual relationships and clinical biomedical issues concerned with “right” courses of action primarily in acute care settings with a lack of attention to ethical issues in public health [32]. Furthermore, dominant bioethical frameworks that focus on individuals and biomedical issues do not address PH ethical concerns adequately [33–35]. Thus, PH ethics is developing distinct from clinical bioethics with a focus on beneficence, respect for persons, and justice [36–40]. Unlike clinical bioethics, PH ethics: [1] concerns populations, public policy and policy structures rather than individuals; [2] places equity at the forefront; [3] includes actors outside the healthcare sector; [4] focuses on prevention of illness and disease, and health promotion [41].

Several authors have proposed health equity as foundational to PH ethics [13, 15–17, 42]. Dan Beauchamp wrote “public health should be a way of doing justice” ( [17] p. 8) identifying that *public health is social justice*. Peter [43] applies Rawls’ approach to justice as fair societies to argue that social inequalities are wrong when they stem from unjust social, political and economic institutions. He states further, “it thus embeds the pursuit of health equity in the pursuit of social justice in general” (p.160). Faden and Powers offer “a non-ideal theory of justice, intended to offer practical guidance on questions of which inequalities matter most when just background conditions are *not* in place” ( [16] p. 30). Their Twin Aim Theory of Social Justice [44, 45] explicates six elements of well-being as criteria for procedural justice at the level of the individual, whilst simultaneously addressing the design and reform of social arrangements to guard against systematic patterns of injustice. Their work sketches out normative ethical guidance for policy makers [16].

As Peter [43] points out, Rawls did not include health as a primary good but did include self-respect as an important primary good and that if certain social positions are devalued “such that people cannot gain a sense of self-respect, then these structures are unjust” (p.167). Jennings [46] draws attention to political theory and relational interpretations of agency, autonomy, and justice as well as values of collectivity, equal respect, parity of voice, mutuality, and solidarity as important to conceptions of PH ethics. Baylis, Kenny and Sherwin’s [15] and Kenny Sherwin and Baylis’ [42] conception of PH ethics emphasizes solidarity and the public good. Using relational theory, these authors see individuals as interdependent and socially, politically, and economically situated. Rather than listing a hierarchy of principles, where independent autonomy is privileged [47], this approach holds competing ethical issues in tension towards the interdependent aim of the public’s health, while recognizing that persons are not all equally situated in relation to opportunities for health [42]. Thus, recognizing the many ways in which persons can be differentially constrained based on different social locations. The PH practitioner’s task is to make visible the impacts that result from policy and healthcare decisions with a view to equitably balancing competing demands amongst differently situated social groups.

However, to develop normative guidance for PH, it is critical to ground ethical theory and perspectives in the everyday ethical concerns that arise for practitioners [48]. Applied ethicists Leget, Borry and DeVries [49] argue that a critical ethical approach integrating empirical research and normative ethical theory can clarify issues and has the potential to set the conditions for supporting real world practice through an ongoing dialectical process. Thus, there is a role for research in shaping the development of ethical frameworks recognizing that these frameworks should inform practice with the reverse also being true. Past investigations of ethics in public health practice have identified issues related to collaboration, priority setting, resource allocation and decision making [50–52]. We found one study of public health decision makers and health equity [53] but none that focus explicitly on public health practitioners and promotion of health equity. A better understanding of the ethical concerns of PH practitioners specifically in relation to their efforts to reduce health inequities is essential to inform frameworks that are relevant and attuned to development of ethical and more equitable PH decision-making and practice.

## Methods

This study is one of four interrelated studies in a broader program of public health systems and services research with an overarching aim to generate knowledge about the integration of an equity lens in PH during a time of PH system renewal in the province of British Columbia (BC), Canada [54]. The PH areas of mental health promotion and the prevention of harms of substance use were the focus of a constructivist grounded theory study to explore the process of how PH practitioners navigate health equity work [55, 56]. As part of this grounded theory, we identified a range of ethical issues in the promotion of health equity. Understanding these issues is an important starting point for understanding the process of navigating health equity work. This study received ethical approval from the University of Victoria, University of Saskatchewan, and six participating Health Authorities (HA) (REB# H11–03359). We obtained written consent from participants.

### Sampling and data gathering

We used purposive and snowball sampling in collaboration with HA partners to identify PH practitioners with PH responsibilities for mental health promotion and prevention of harms of substance use in their work. The sample consisted of 32 participants with whom we conducted 29 semi-structured interviews (face-to-face and phone) and one focus group of 3 people. We developed the interview guide for this study, and it is available as a supplemental file. Participants represented five regional HAs and one Provincial Health Authority. Participants had, on average, 10.26 years of PH experience. Registered nurses (RNs) were the majority of participants [25] and all but one had post-secondary education. Interviews averaged 60 min and were recorded and transcribed verbatim by an experienced transcriptionist and verified by the study research assistant.

### Data analysis

We employed constant comparative analysis, a method foundational to grounded theory and an accepted approach to qualitative enquiry [57–59]. This method involves detailed coding to develop concepts and relationships among the codes by comparing incident-to-incident, incident-to concept, and concept-to-concept to take the analysis from the “ground” up through higher levels of abstraction [60]. Our team of five researchers (four faculty and one research assistant) generated a set of inductive codes for the coding framework from line-by-line coding of the initial three interviews. The research assistant continued coding the interviews line-by-line using the initially developed framework and adding to the coding structure using NVivo software. The team conducted in-depth analytic discussions to compare incidents within the same interview, across interviews, and over time to establish relationships and differences between incidents and concepts [55, 60]. We employed memoing, diagramming, and reflexivity in the conceptual development of the key themes and to enhance rigor. The eventual grounded theory (GT) is reported elsewhere [56]. In this paper, we describe in depth one element of the grounded theory - the specific ethical tensions identified by PH practitioners that arose from their practice promoting mental health and preventing harms of substance use.

## Results

Participants identified four systemic ethical tensions: [1] biomedical versus social determinants of health agenda; [2] systems driven agendas versus situational care; [3] stigma and discrimination versus respect for persons; and [4] choice and autonomy versus surveillance and social control. In describing these four tensions, we lay out the full range of ethical issues expressed by participants as a group. The extent to which individual participants were aware of or described these tensions varied across participants. While some practitioners expressed a high degree of awareness of a range of issues, other practitioners were less aware of the broad range of ethical issues related to health equity work. So, the lens for viewing what constituted an ethical issue in health equity work varied across participants. As described elsewhere [56], PH practitioners with a critical public health ‘lens’ were more likely to recognize and experience ethical tensions. For each of these ethical tensions, we describe the underlying values conflict and identify the ethical concerns from the perspectives of PH practitioners. Although we present each of the four areas of ethical tensions separately, they are interrelated, and PH practitioners often must simultaneously navigate these issues to promote health equity.

### Theme 1: the health equity curse: biomedical versus social determinants of health agenda

Participants identified a primary area of ethical tension as the dominance of a biomedical agenda that obscured the PH focus on health equity, with a subsequent lack of focus on the social determinants of health and systemic responses to reduce health inequities. They defined the biomedical agenda as the dominance of acute care priorities with a consequent emphasis on the treatment of disease, illness and injury for individuals rather than prevention and promotion. The following participant working on an integrated outreach team describes:

*We’ve got the swing how to get people tested to make sure that the medication is working for them. So the medical system is actually quite good, quite slick. But in terms of the support for the other parts of their sort of hierarchy of needs, the housing, the food, you know all the stability that goes along with, or instability that goes along with poverty, I think we are still a long way from sorting that out.* (S4–29)

Participants observed that within a biomedical system, there is a lack of understanding and valuing of PH work especially the work of prevention.

*It’s really hard to say we prevented this mom from harming her baby, or we prevented this mom from having a postpartum psychosis and going into a hospital … it’s really hard to say we prevented something from happening. And so, you know, money and hospitals, people can see when they’re voting or putting money into the healthcare system, they can say, okay this x-ray machine does x-rays that prevents pneumothorax, which prevents death. Right? So that’s an easy thing for people to see, but in prevention and health promotion, it’s really hard to say, you know, having these clinics will prevent something from happening because the outcome should be nothing. And it’s really hard to prove nothing.* (S4–04)

Furthermore, participants highlighted that even within PH what seemed to matter was communicable disease prevention and a focus on secondary prevention with less attention to primary prevention or health promotion such as described by the participant above.

*And so the (name of organization) says that they’re into health equity, and they say they understand social determinants of health, but if you look at everything they do and all of their work plans and stuff, they’re all about some bugs and viruses, and emergency, you know, Ebola responses … ..That seems to be the level of where we sit with health equity and they don’t know how to talk about- or they don’t publicly talk about what health has to say about the … the systemic stuff that we have, our policies that create health inequity.* (S4–02)

While participants recognized that the health system was not solely responsible for addressing the determinants of health, the lack of value for the PH role and focusing upstream on the root causes of health inequities were often described by participants as unimportant to health systems. This same participant describes the daily ethical challenges of working in health care organizations that do not embrace health equity:

*We’re ethically challenged every day because we work in health care in a place where people don’t have adequate services, don’t get treated well in the system, don’t have proper housing. So, sort of a different level for me. I was just going to say … . once you start seeing the world through a social determinants lens, it’s like you’re- you can never go back and it’s a bit of a curse. It’s not easy.* (S4–02)

Other participants also shared that once you saw the world through a health equity lens it raised more ethical challenges than if you did not, because it means living with the moral discomfort of knowing what is needed but being unable to act. They acknowledged that sometimes it was easier for them and their colleagues not to hold a health equity lens because accepting the status quo reduces discomfort. This paradoxical “health equity curse” included knowing that clients, families, groups, communities and populations are experiencing deficits in resources for health but their needs do not fit into the dominant model of biomedical care.

This lack of access to resources for health was a fundamental ethical issue and experienced as morally distressing, as described by the following participant:

*Because you know, you’re stuck … .you can’t give people a better house … ..You can’t get them a sink, you can’t give them the basic needs, right? So you are, you’re very torn and almost feel guilty at the end of the day when you go home and you think, like “God,” you know? You stand in question of what you have and what you need, and what people need in society.* (S4–20)

Working within a healthcare system that fails to act on the determinants of health weighed heavily on the PH practitioners in our study. They felt that they had few, if any, resources available to address determinants of health or the structural causes that produced health inequities or the subsequent distress associated with being aware and unable to act.

### Theme 2: procedures, checklists and checkboxes: systems driven agendas versus situational care

Participants highlighted how the pressure of meeting systems requirements drove PH work rather than the situational needs of clients. Participants pointed to systems requirements such as procedures, guidelines, checklists and checkboxes as the drivers of their work and ultimately actions/inactions taken to promote health equity. One participant described:

*You know, public health is so indoctrinated with policies and procedures and guidelines and charting, and again, that often gets taken up with, you know, what’s being delivered from above into how we do our work, So again, it’s not really about the clients themselves and the work with them, but it’s about the criteria put out by [Health Authority].* (S4–12)

In particular, PH practitioners described how systems requirements, based on standardized assessments rather than structural or situational factors were prioritized when it came to determining eligibility for services and programs. One participant stated:

*Certain mothers quote unquote “qualify” for a home visit due to some varying risk factors. And is that an equitable way of treating our population? Because it leaves out that aesthetic way of knowing about that person. You know? Saying “I just have this feeling that this mom needs a visit” or “just from her tone of voice, I think she’s not telling me she’s depressed but I sense something” so I go out and visit and sure enough, there’s several different things going on*. (S4–18)

In addition to program eligibility criteria that allowed little room for clinical assessment of situations, participants described the ethical issues of working with checklists/checkboxes, procedures, and guidelines rather than focusing on the person and their context.

*So what I mean by that is probably in this office if we were to do ideal nursing work or ideal support, family support work, we would be able to call all the moms and ask them what they wanted from us and be able to implement that whether it’s going out to see them in their homes, or taking them to, you know, the store to buy proper food, you know, helping them, whatever they wanted, whatever they felt that they needed at that time to meet where they were at. If we were able to do that without constraints of resources and um checklists and things like that, … ..So I think for us, all our ethical dilemmas come from the facts that we work on the ground very differently than what the people who create our resource pool and our jobs, and our job description work from.* (S4-04).

The examples above also highlight a move away from universal to targeted programs with a focus on standardized criteria for assessing risk. One participant describes the evolution of this shift.

*You know there’s always a nurse available that if a parent had been discharged with a new baby, they would get a home visit to make sure that things are going well, you know, to do an assessment on their mood. So the universal program over the last number of years is getting more streamlined into more targeted populations, the higher risk group or the higher priorities is how they term it in public health. So that, the universal approach, is kind of shifting a bit, looking at budgets, you know, how to invest your money, right? But always I feel that with the thought of universal approach, a lot of people get kind of lost - because it’s not always obvious that there’s issues, right?* ( S4–12)

Systems requirements related to program eligibility, procedures, checklists and checkboxes and shift from universal to targeted programs shift the focus away from promoting equity in that resources cannot be based on assessment of need. Some practitioners pointed to the mantra of patient centered care as a health system priority but with little attention to the social conditions that impact individual health reflecting a value of individualism/ neo-liberalism. Thus, there is a tension between systems driven agendas in which the focus is on meeting the demands/needs of the system and situationally driven care in which individuals and their needs are understood within a set of social circumstances.

### Theme 3: systemic stigma and discrimination versus respect for persons

Participants described stigma and discrimination as pervasive within health care systems. They described witnessing various forms of stigma related to mental illness, substance use, addiction, HIV, blaming and criminalizing of people experiencing health inequities.

*I find there’s more judgement. You know … not having the same kind of emphasis or compassion, or understanding of the complexities of health inequities, you know, and the determinants of health, even though that is part of the lens in public health, there’s still sort of … there’s a certain attitude of like they choose just for themselves.* (S4–12)

The quote above highlights a dominant understanding that it is the individual who is to blame (e.g. they choose this for themselves) rather than a recognition of systemic inequities. The participant below describes how this plays out specifically related to mental health and substance use.

*Oh we won’t treat you if you’re using and if you’re mentally ill … maybe it’s because of your use, so therefore we won’t deal with you, I think that really reflects our society’s attitudes, about, probably our state- about how we feel about mental health and how we feel about addiction, right? So if you’re so … I don’t know, you know, ‘lazy’, or ‘unorganized’ or ‘undisciplined enough’ to be using something, we’re not- so this is an underlying theme with addiction: you know, you’re not -you’re just a drain on our system and so, you know, you’re wasting bed space here because you’re addicted to something … So we want you to get it together before you come back … So there’s that serious underlying theme that threads through how, I think, our society sees people who use drugs. And then, you know, so if they come in using and with mental health, that kind of gets layered into how they’re treated.* (S4–02)

While participants recognized stigma of mental health and substance use as in the example above, they were less likely to name intersections of stigma with various other forms of discrimination related to ethnicity, sex and gender.

*You know, there’s certain gaps for instance for the First Nations population who don’t live on reserve, they can access our services, right? But, you know, there’s just always, maybe not as comfortable to walk into our building that’s very clinical and very institutional feeling – it’s a very old building. You know, big counter, so I mean I think that can be a barrier for people feeling comfortable to access the services.* (S4–12)

Although this PH practitioner did not directly name racism or link the ‘institutional feeling’ to a colonizing history, racial discrimination compounds other stigma related to mental health and substance use. Participants did at times identify sex and gender as areas of discrimination but did not necessarily recognize or identify the intersections of various forms of stigma and discrimination.

As a result of various forms of stigma and discrimination, participants described healthcare systems as producing mistrust and affecting health care experiences of populations they were working with.

*And because they’ve probably been treated in the past, they’re not wanting to access service and they mistrust now … .The majority of my clientele that I work with will not, and I’ve never seen this before, will not go to the hospital. And I kid you not, until it’s almost too late or too late. I’ve never seen that, because of how they’ve been treated.* (S4–25)

Participants described how their clients’ concerns were often dismissed outright and the challenges related to system processes such as navigating through bureaucracy, filling out multiple forms and getting through gatekeepers was daunting, creating ethical concerns related to the personal capacity and energy of clients and practitioners to work to access a system that is highly stigmatizing and limited in what can be provided. For example, one participant described the work of carefully choosing terminology in documentation to favorably present a client so that they could get access to housing and described this as ‘*fudging it*” rather than seeing this as a way to reduce stigma knowing that housing was scarce commodity in the community.

Several participants discussed how the line between practitioner and client experiences is not so distinct. Some participants self-identified as having past problematic drug use, being gender non-conforming, having experience with mental health issues, or having family members or loved ones in need of mental health or substance use supports. One participant described how their identity as queer was not recognized as an asset in the workplace but rather something that they had to manage carefully in terms of who they shared this information with. Finally, because the work of PH practitioners brought them close to groups that are so often stigmatized, they were found themselves personally impacted by stigma, *“I think the work that we do is also stigmatized. Like, our clients are stigmatized for their health and social status and we are stigmatized for working with them”* (S4–20). Thus, having to navigate stigma and discrimination on multiple fronts for themselves and their clients. However, there was seemingly little appetite to address systemic stigma within organizations.

*My agency ... they say they care about these issues [of equity], but if we start talking about them too much, they tell us to not talk openly about it. Yeah. Like a few of us will get quite fired up every so often about how they profile groups, and then, you know, say “These people are more at risk” and they stigmatize them sort of there, or don’t look at all the complexities that go into why that group, you know, is more vulnerable.* (S4–02)

For these practitioners, discussion was stifled leaving sources of inequity unaddressed and continuing to operate in the very systems meant to provide care.

### Theme 4: trust and autonomy versus surveillance and social control

The context of relationships between practitioners and clients was one of mistrust due to systemic stigma and past negative experiences in healthcare. Consequently, participants indicated that building and preserving trust and autonomy were priorities that sometimes came into conflict with organizational or legislated demands that required measures of surveillance and at times social control.

Participants particularly noted concerns related to trust and autonomy around maintaining confidentiality and consent regarding communicable disease reporting to protect the public. Participants shared how navigating STI reporting requires a nuanced approach to keep clients engaged in care and meet population health mandates. It takes time to build trust, learn details and assess risks in a situation as well as decision making about how to reduce both individual and population risks. One practitioner described working with a client who was positive for HIV and she had not told her partner.

*Only a few hours ago we were faced with this ethical issue where one of our clients who comes up from time to time, where we know that she has an ongoing relationship with someone who isn’t aware of her HIV status. And so that’s always a bit of an issue ... but they aren’t sexually active, so it hasn’t been a big concern to us that he doesn’t know. But he said that yesterday he was picking her up and then he was poked with a needle. And so suddenly I’m thinking he needs to know so he can access care, he should be offered post exposure prophylaxis and the window is so short for that. But we can’t inform him and break her confidentiality. I wonder if we can find her to talk to her and let her know, like “hey this is what he told us. Can we work with you at all to disclose?” . . . So I was sort of sitting here thinking “I can’t not do anything” . . . And I think really feeling the pressure of it because of it being this short time frame where we if we can get anything happening, we need to get it happening now.* (S4–23)

This exemplified how practitioners work to preserve trust as well as being finely attuned to their clients and their clients” particular situations as they worked to navigate their obligations in the face of possible risk to the public. As our participants described, approaching disclosure in a client led way was emotionally intense and required persistent engagement, and ongoing assessment. Acting prematurely might cause the client to disengage and lose trust in the practitioner and then increasing risks for population health. Thus, knowing when it was appropriate to break confidentiality to disclose private information was a delicate relational dance in which the practitioner had to balance the relationship with client and the health of others as circumstances unfolded. Although a different form of surveillance, the practitioner below describes being requested to check up on a client.

*And the times where I feel like my ethics have been compromised is where I’ve been asked to have quite a specific follow up. Like, you know, one example would be to call the doctor to make sure that the client attended for a baby checkup. Or something like that. For me, .., if that was an agreement I had with the client already personally, I would feel okay about that. But for me as maybe we’ve never even met, that feels like policing and that feels unethical to me.* (S4–21)

This participant highlights that being asked to check on someone is a form of policing in healthcare that feels unethical. Other participants described ethically challenging situations as knowing when to call the police or child protection, knowing that such calls would bring in systems of social control and work against the hard-fought earning of trust. Participants described being in the position of working to preserve trust and autonomy with their clients and attempting to manage surveillance and social control to prevent further inequities. We would note that a focus on managing surveillance and control takes the emphasis away from providing support and access to resources that can promote health equity.

### Strengths and limitations

There are several limitations of this analysis. First, it specifically focused on issues experienced by PH practitioners in promoting mental health and preventing the harms of substance use at a specific point in time. Ethical issues may be different in other PH core program areas. Yet, this may be an area of PH work where a lack of attention to social determinants of health (SDOH) was more readily visible and apparent but at the same time may not reflect health systems programs to connect people to the SDOH or programs within health authorities that address issues related to SDOH like housing. Second, this study took place in one provincial geographic context in Canada, representing rural to urban settings with different systems of health care delivery across six different publicly funded Health Authorities. Reporting to the provincial Ministry of Health, each Health Authority delivers the same public health services but tailored to their context. However, this may also be a strength and contribute to the opportunity to extend these findings to other contexts.

## Discussion

In this paper, we have sketched out systemic ethical challenges in PH practice related to the promotion of health equity. We specifically outline four systemic ethical challenges that arise in PH practice related to the dominance of biomedicine, bureaucratic systems, systemic stigma and discrimination, and potential systems of surveillance and control. All of these shape PH providers’ interactions with clients and affect their ability to promote health equity. The dominance of biomedicine in the health care system focuses action on treatment of disease for individuals leaving little space for public health and little attention to the broader social determinants of health or more simply the conditions in which people live and work. Systems requirements, often infused with the values of biomedicine and individualism (such as procedures and standardized checklists) do not account for the unique situatedness in which individuals, groups and communities are positioned. In the current health care system dominated by biomedicine and bureaucratic approaches to care and the presence of stigma, it is often difficult and challenging for PH providers to meet obligations related to health equity leaving them with the burden of unmet needs and feeling like health equity is a curse. This study provides insights into health equity issues in public health that are only briefly mentioned in others studies of public health ethics issues noted at the beginning of this paper.

Notable in the ethical concerns of PH providers is the pull of biomedicine, neo-liberal and individualist discourses that obscure the broader social and often structurally violent conditions that produce vulnerability to health inequities. Similarly, in a systematic review of literature on health equity, Farrar and colleagues [61] identified that capitalism, biomedicine and difficulties with collaboration impact the ability of public health to advocate for health. In fact, Smith [53] found that PH decision makers were uncomfortable and shied away from issues related to justice and power. Participants described having to practice within health systems that drift to targeting behaviors of individuals, groups, and populations rather than recognizing social, economic, historical, and political risks, and social conditions that impact health. Despite the growing evidence that targeted behavioral approaches have limited utility for groups experiencing disadvantage [62, 63] are ineffective [64] and may, in some cases, even widen inequities [65], lifestyle and behavioral approaches still dominate within Canadian PH policy [21, 64]. In fact, much of what participants are calling for here in relation to assessing and providing resources based on need is aligned with proportionate universalism, an approach where health actions are universal but provided in proportionate to level of disadvantage [66].

Stigma related to illicit substance use, homelessness, mental illness, HIV, Hepatitis C, often intersect with forms of discrimination including racism, classism, and gender bias [8]. These findings broaden the understanding of stigma as an ethical issue in healthcare beyond association with disease conditions to encompass poverty and substance use stigma [67]. Stigma and discrimination contribute to social exclusion, limit access to resources for health and exacerbate health inequities. It is clear from our findings that stigma is pervasive in healthcare reflecting unjust structural arrangements limiting the achievement of health equity. Of note, participants often spoke to one but not multiples sources of stigma. It is not clear whether this is due to the complexity of multiples stigmas and discrimination and/or lack of knowledge about how various forms of stigma and discrimination compound creating even greater inequities for some. Furthermore, it is of serious concern that participants felt that their attempts to address systemic stigma within healthcare are stifled and that they experience stigma by association, also known as courtesy stigma [68, 69].

All PH practitioners in our study described the negative effects of seeing firsthand or hearing stories from their clients about the challenge of living with health inequities. They bore witness to the interface of clients with the healthcare system and the inability of such systems to address health inequities. Thus, what became clear is that PH practitioners’ call to act is not an abstraction but comes from their professional obligations and from working with, alongside, and in communities experiencing health and social inequities. Much has been written about moral distress in acute care with less attention to moral distress among PH practitioners [70–72]. What is strikingly similar is the degree to which PH practitioners are bearing witness to systemic issues over which they have little control (e.g. structural conditions) and as a result feel powerless to assist their clients even when health equity is articulated as important and expected [25].

Reducing health inequities must include actions that: [1] improve the conditions of daily life; [2] tackle the inequitable distribution of power, money and resources; and [3] measure and understand the problem and assess the impact of action (Commission on the Social Determinants of Health, 2008). As Jennings [46] observes, both political and moral theory are important to the future of public health ethics. Given the ethical issues identified in this study, we could not agree more. Addressing biomedical, bureaucratic and individualist ideology as well as stigma and discrimination is inherently political. Jennings [73] expands on the need for public health ethics to be informed by concepts of relational solidarity and care. More specifically, Baylis, Kenny and Sherwin’s [15] relational conception of PH ethics resonates with the issues related to health equity in PH practice. Relational perspectives are reflected in the narratives of participants, highlighting the importance of understanding how individuals are situated as part of health equity work. This is directly aligned with health equity perspectives that focus on the importance of social, historical, political and economic positioning for understanding individual and community access to social determinants of health [1]. We would highlight that the ability to recognize ethical issues in practice is part of the competencies of public health providers and that these competencies include naming ethical issues as well as identifying appropriate courses of action. In the view of these findings, achieving such competencies is more so a curse than an achievement in health systems that do not operationalize values of health equity.

## Conclusion

The ability to enact PH as social justice is largely constrained within a system that privilege biomedicine and bureaucracy even when there are commitments to health equity. As well, stigma and discrimination are embedded deeply within health care systems and practitioners are constrained in their ability to disrupt these patterns of devaluing and exclusion by the system itself. Critically important to working with individuals and groups experiencing health inequities is the ability of practitioners to think situationally and to preserve trust in relationships, but this often comes into conflict with duties to protect the public as well as societal pressures to surveil and exert social control. As with any complex challenge, there needs to be multiple avenues to action to transition to equity-focused health care systems. As with any complex challenge, there needs to be multiple avenues to action to transition to equity-focused health care systems. Decision makers and public health practitioners need to examine and track the process of operationalizing values of health equity as values alone are not enough [74]. Setting benchmarks and reporting on progress can help to alter the culture of a biomedical focused system and provide support for practitioners to encourage shifts toward equity-oriented systems. Additionally, all health care practitioners need improved education and support in application of structural competencies and encouraging practitioners in critical decision-making rather than bureaucratic checklists. We highlight that the frameworks and perspectives of relational ethics offer a more promising approach for recognition and attention to health equity issues in public health.

## Supplementary Information

**Additional file 1.** Interview Questions S4 – Supplemental File. Interview transcripts. Semi-structured interviews were guided by the use of the interview questions for both the individual and focus group interviews. Interviews were audio recorded and transcribed verbatim.

## Data Availability

Data are not available due to the sensitive and confidential nature of the data.

## Abbreviations

PH: Public health
RN: Registered nurse
SDOH: Social determinants of health
